# Croco Eye Technique: Mucous Retention Cyst Excision with Immediate Open Sinus Lift—A Retrospective Cohort Study

**DOI:** 10.3390/jcm13113293

**Published:** 2024-06-03

**Authors:** Radosław Jadach, Karolina Osypko, Kamil Nelke, Adam Nowicki

**Affiliations:** 1 Private Practice, ul. E. Horbaczewskiego 53A, 54-130 Wrocław, Poland; radek.jadach@gmail.com; 2 Dental Salon, Oral Surgery Academy, ul. E. Horbaczewskiego 53A, 54-130 Wrocław, Poland; 3 Maxillo-Facial Surgery Ward, EMC Hospital, Pilczycka 144, 54-144 Wrocław, Poland; kamil.nelke@gmail.com; 4 Diamante Clinica Dental Clinic, Sportowa 48 A/C, 59-300 Lubin, Poland; adamusnowicki@gmail.com

**Keywords:** croco eye technique, sinus lift, sinus floor elevation, mucous retention cyst, biopsy, excision, retrospective cohort study

## Abstract

**Objectives**: A mucous retention cyst is a common, asymptomatic lesion that may cause complications during or after the sinus lift procedure. The goal of this study is to assess the effectiveness of the Croco Eye Technique (CET), which allows simultaneous excision of the cyst and sinus floor elevation. **Methods**: The technique was thoroughly described in two versions, and the group of 33 patients was analyzed. Patients who qualified for this procedure had insufficient alveolar ridge height, and their CBCT showed radiological images typical for retention cysts. Analyzed parameters included the version of CET, demographic data, anatomical parameters, intraoperative complications, recurrence of the cyst, success rate of the sinus lift and implants, and the follow-up period. **Results**: Out of the 33 cases, 9 were of the primary version (27.27%) and 24 of the final version (72.73%). The average height of a retention cyst was 24.05 mm, with the average alveolar ridge height of 1.86 mm. In three cases (9.09%), implants were placed immediately. The prevalence of uncontrolled Schneiderian membrane perforation was reduced from 55.56% to 4.17% between the primary and final versions. The cyst’s recurrence rate was 3.13%. The implant survival rate was 100%. The mean follow-up period was 48.625 months (max 110 months). **Conclusions**: The Croco Eye Technique, despite the perforation of the Schneiderian membrane, enables successful sinus lift and implantation with a success rate of 100%. Excision of the retention cyst, which is the cause of perforation, allows for limiting the risk of the cyst’s recurrence.

## 1. Introduction

Retention cysts are quite common (up to 28.6% [1]) asymptomatic lesions [2] in the maxillary sinus that often do not require any surgical intervention as a standalone procedure. The problems may start when a patient is qualified for an open sinus lift procedure due to low alveolar ridge height. Any presence of a retention cyst in the sinus is an obstacle that disturbs an otherwise predictable treatment plan [1].

Mucus retention cyst develops when the seromucous duct is obstructed and dilates due to continuous activity of the seromucous gland [2,3]. The duct may be obstructed as a result of odontogenic inflammation (such as periapical lesions [1]) and seasonal changes [4]. Also, certain anatomical variations such as ostium diameter, its height from the sinus floor, septal deviation, accessory ostium, and concha bullosa may influence the retention cyst’s occurrence [5].

The main goal of the Croco Eye Technique (CET) is to simultaneously remove the mucous retention cyst while causing as little damage to the Schneiderian membrane as possible and to successfully perform a sinus lift procedure.

This technique eliminates the need for an endoscopic approach or a delayed second procedure just for sinus floor augmentation. Moreover, it may be conducted without any specialistic equipment or materials (other than these for standard sinus lift procedures) by any trained clinician.

The goal of this study is to analyze medical documentation and determine the outcomes of this technique in a group of 33 patients who underwent this type of procedure over the last 9 years. The initial hypothesis states that this technique enables definitive cyst excision and implant placement within the augmented area with no adverse effects.

## 2. Materials and Methods

Study design and setting

The first part of the article concentrates on describing the CET procedure in a detailed, step-by-step manner. As this technique was modified over time, we wanted to present three cases: the primary version, the final version, and a variation of the final version upgraded with a surgical guide for window osteotomy. Authors agree that the classic final version (presented in case 2) is the recommended one, as it is the safest and most universal.

While the first online mention of the technique was in 2016, throughout the years, it has been modified. We present our refined Croco Eye Technique and a subset of patients using our modified technique.

The second part of the article summarizes the clinical data collected from February 2015 to April 2024.

Participants

All the patients qualified for this procedure needed to meet the inclusion criteria:

- Insufficient bone height for direct implant placement or closed sinus lift procedure (less than 4 mm of alveolar ridge height);
- Radiological image of retention cyst (homogeneous “dome-shaped”/“rising sun” solitary radiopaque mass located at the floor of the maxillary sinus);
- The lesion in the sinus had to be asymptomatic;
- Unobstructed (radiologically) airflow between the maxillary sinus and the nose;
- The patient had to be willing to have implants in this area instead of a dental bridge or a removable denture.

The exclusion criteria were as follows:

- Sinus pathologies: osteomeatal complex obstruction, chronic rhinosinusitis, and radiological image of solid sinus tumors;
- Uncontrolled diabetes;
- Substance abuser;
- Nursing or pregnancy;
- Aminobisphosphonates treatment;
- Untreated periodontitis;
- Full mouth Plaque Index > 25%.

Variables and data sources

The analyzed sample contained 33 patients, 18 males and 15 females, with an average age of 49.33 years (minimum 29, maximum 79 years).

The analyzed parameters were as follows:

- Version of the CET (primary vs. final);
- Demographic data (sex, age);
- Anatomical parameters before surgery (height of the residual cyst, height of the alveolar ridge, presence of anatomical difficulties);
- Details about surgery (uncontrolled perforation and its suturing, type of bone grafting material, immediate implantation, graft dislocation to the sinus);
- Assessment after at least 6 months (radiological success of sinus lift, recurrence of the retention cyst);
- Assessment of success of reconstruction on implants at least 12 months after implantation;
- Follow-up period.

Measurements and radiological diagnostics were conducted in an imaging system dedicated to CBCT radiographs (0.2 mm voxel, FOV: 14 cm diameter × 8.5 cm height, GXCB-500 HD, Gendex, Hatfield, PA, USA/0.1 mm voxel FOV 10 × 10 cm CS 8100 3D, Carestream, Rochester, NY, USA). Even if a patient had an OPG performed, it was considered an additional examination. Moreover, only the retention cysts in the area of the future sinus lift were treated.

The data were collected by analysis of patients’ medical history, CBCTs, photographic documentation, and with agreement from the patients to use their cases for scientific purposes. The study size was determined by collecting all cases of patients who underwent this procedure from the beginning of February 2015, as these cases were thoroughly documented. All the results are accumulated in Table 1.

The follow-up strategy focused on the following:

- Analyzing the latest CBCT of a given patient, with a special focus on the presence/absence of radiological images typical for retention cysts (which would suggest the cyst’s recurrence) and any radiological signs of periimplantitis;
- Analyzing medical history through the prism of inflammation (e.g., pain, swelling) or any functional problem around implants.

We also contacted all patients whose latest appointment was more than 12 months ago and invited them for a free check-up with control CBCT, although not all of them came.

The study was conducted under STROBE guidelines.

### 2.1. Case 1—Primary Version

**Figure 1:**
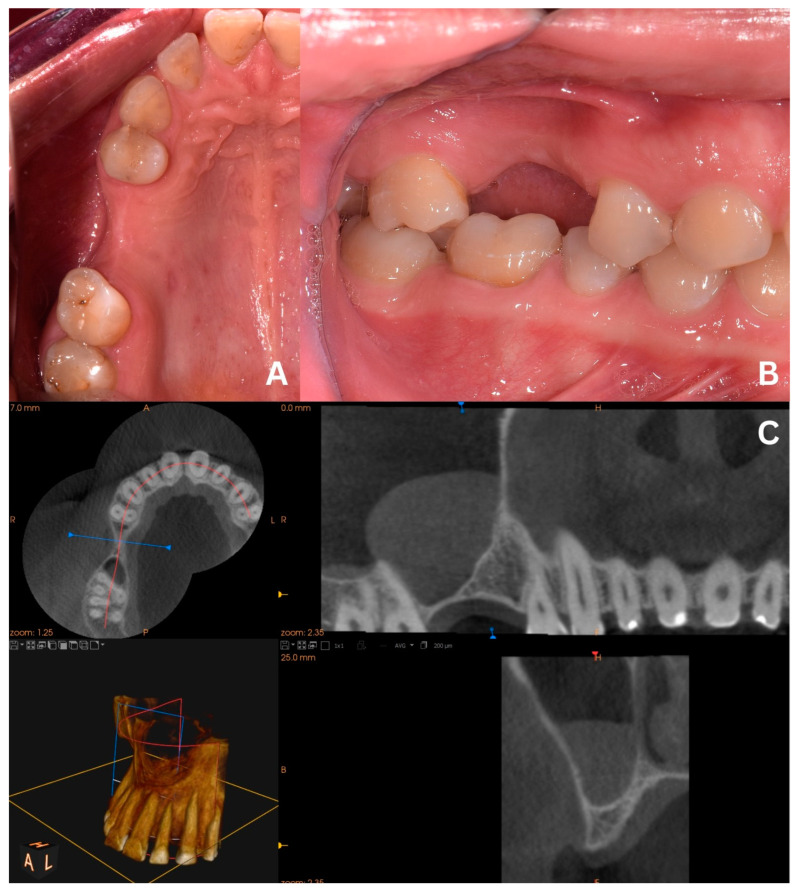
Clinical situation before the surgery. Occlusal view (**A**), lateral view (**B**), and CBCT scans with a dome-shaped radiopaque lesion in the sinus and insufficient bone height that requires sinus lift before placing implants (**C**).

This case sets a proper example of the primary version of the CET that has been conducted on a patient qualified for open sinus lift before implant placement in positions 16 and 15 (FDI numbering system) (Figure 1A,B). CBCT unveiled a homogeneous “dome-shaped”/“rising sun” solitary radiopaque mass located at the floor of the right maxillary sinus, precisely above the region of the planned sinus lift (Figure 1C). The radiological image, class III B by Di Girolamo classification [6], combined with the absence of symptoms, suggested a mucous retention cyst or pseudocyst of the maxillary antrum [2]. Although in such cases, Di Girolamo et al. suggest endonasal sinus surgery (ESS) before sinus lift due to possible difficulty in sinus floor elevation and risk of ostium obstruction, our article provides a solution to avoid ESS and prolonged treatment without compromising the sinus lift procedure and the final outcome.

The procedure was conducted in local anesthesia (articaine 4% with adrenaline 1:100,000) with premedication: amoxicillin + clavulanic acid 2 g (2 × 875 + 125 mg), nimesulide 200 mg, and paracetamol 1 g.

**Figure 2:**
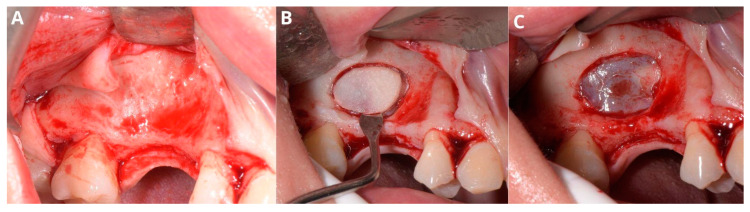
Creating access through the lateral window. Elevation of the full-thickness flap (**A**), osteotomy with piezotome, and removal of the “bony lid” (**B**), undamaged Schneiderian membrane with visible vasculature (**C**).

A wide mucoperiosteal flap designed with a sulcular incision and one vertical releasing incision distally from tooth 17 was elevated to unveil the lateral wall of the sinus (Figure 2A). An ovate osteotomy window was opened with a piezotome (Guilin Woodpecker Medical Instrument Co., Ltd., Guilin, China), and after gentle removal of the window (Figure 2B), the Schneiderian membrane with vascular branches was visible (Figure 2C).

**Figure 3:**
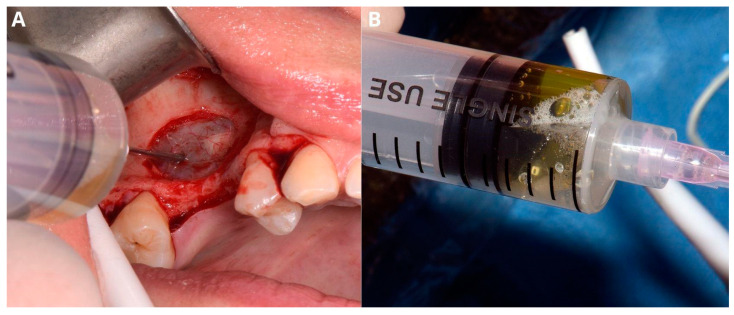
Aspiration of the fluid from the retention cyst. The needle punctures the cyst in the center of the bone window (**A**), which facilitates management of the future perforation; yellowish color of the retention cyst’s content (**B**).

The next step was a biopsy. The cyst wall was punctured through the Schneiderian membrane in order to aspirate and check its contents and to reduce cyst dimensions (Figure 3A). The authors recommend using a 1.2 mm diameter needle in case of dense mucus or pus. Moreover, aiming between visible major vascular branches limits the risk of excessive bleeding and helps keep the operational field clean. Using a 1.2 mm needle results in Scheiderian membrane perforation; therefore, the biopsy point should not be near the edge of the osteotomy window, as managing the perforation requires enlarging the window [7], which may be easily avoided by aiming in the central area. The biopsy may not be successful at the first attempt, as the position of the cyst differs in the patient’s horizontal position during the operation. Aspirating the yellowish mucinous liquid is pathognomonic for a sinus cyst [7] (Figure 3B).

If the piston of the syringe recoils and the syringe remains empty, then we ought to suspect a tumor and follow the steps below:

- Repeatedly pull the piston to collect some of the cells to the needle;
- Take the needle out of the sinus and detach it from the syringe;
- Pull back the piston to fill the syringe with air;
- Plug the needle back;
- Blow the needle’s contents onto the glass plate;
- Preserve the sample with CitoFix (or another solution for fixation and transport of cytological samples) and send it for histopathological examination.

It is recommended to close the wound and postpone the sinus lift procedure unless the surgeon is skilled and experienced and is capable of collecting a section of the tissue rather than taking a fine-needle biopsy.

**Figure 4:**
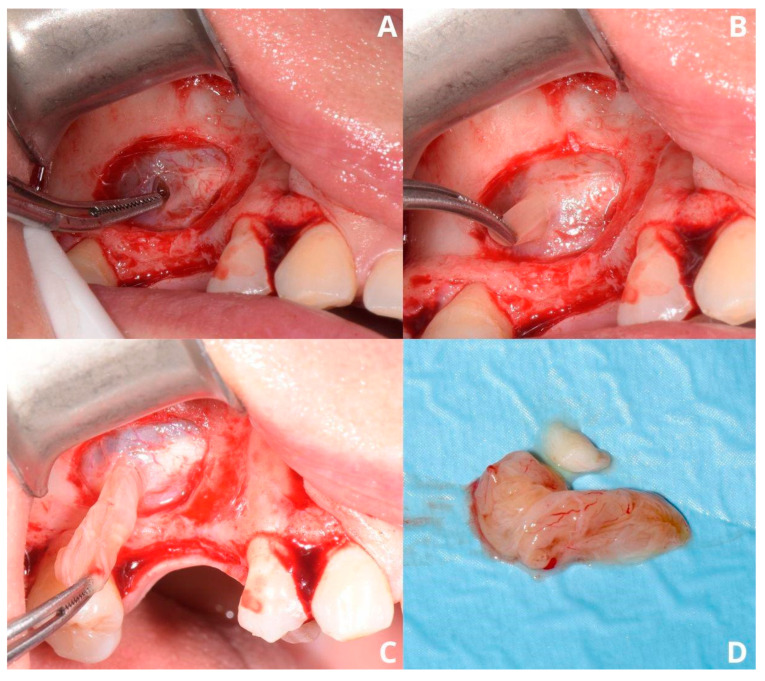
Removal of the cysts’s lining. Two layers of the Schneiderian membrane are visible: periosteum and mucosa (**A**). Gentle removal of the lining (**B**,**C**) and its remains (**D**).

After draining the cyst, its lining needs to be removed in order to prevent recurrence. Failure to remove the whole lining should not be a concern, as high damage and perforation will unlikely refill nor cause recurrence. As the cyst residue is loose, it may be caught with narrow surgical suction. It is not recommended to try to catch the cyst’s lining with a tweezer, as it condemns the surgeon to open the tweezer within the perforation of the Schneiderian membrane and needlessly widen it (Figure 4A). Another technique of catching the cyst is hooking a needle, i.e., a 0.8 mm diameter needle. Previously used 1.2 mm diameter needles are too rigid and may break after bending. Afterward, the cyst may be gently detached (Figure 4B–D) using two pairs of tweezers: one as a security measure to hold it and the second for drawing the cyst in different directions.

Histopathological examination confirmed retention cyst. No evidence of neoplastic pathology was confirmed.

**Figure 5:**
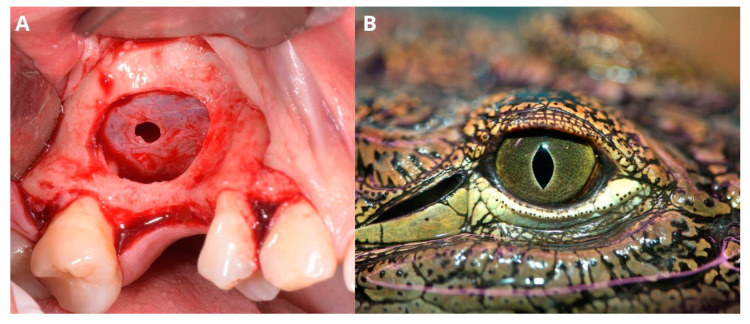
Perforated Schneiderian membrane (**A**) resembles the eye of a crocodile (**B**) (photography purchased via Canva Pro (Canva Pty Ltd., Perth, Australia)).

The osteotomy window and Schneiderian membrane perforation in the central area resemble a crocodile eye, which gave the name of the technique (Figure 5).

**Figure 6:**
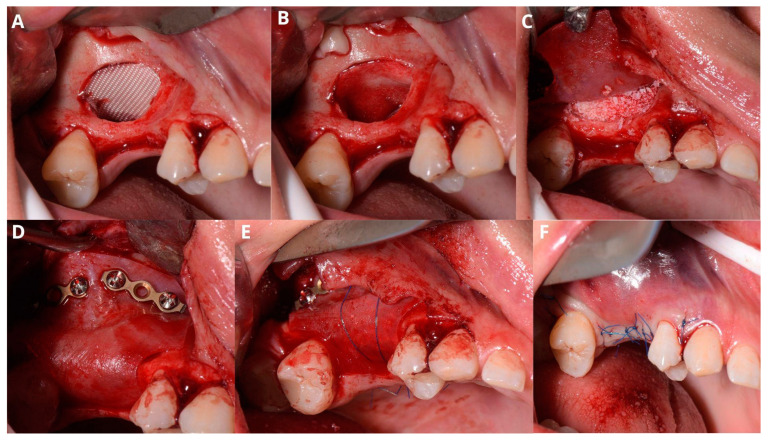
Completing the sinus lift. The first layer that was put under the Schneiderian membrane was oxycellulose (hemostatic material BloodSTOP™ iX (LifeScience Plus, Mountain View, CA, USA)) (**A**). Then, a thick, shape-retaining collagen membrane (**B**) with a xenograft was placed below it (**C**). The second membrane (to rebuild the bone horizontally) was fixed with two osseofixation plates (**D**) and sutured to the palate soft tissues (nylon 5-0) (**E**) to obtain tension-free contact with the flap’s edges. Final suturing was performed with nylon 5-0 (**F**).

After elevating the Schneiderian membrane from the sinus floor and palate wall, the perforation was covered with the hemostatic material BloodSTOP™ iX (LifeScience Plus, Mountain View, CA, USA) (Figure 6A) and collagen membrane Osseoguard Flex (Zimmer Biomet Dental, Biomet 3i, Palm Beach Gardens, FL, USA) (Figure 6B) steeped in I-PRF. The material used for sinus lift was porcine xenograft (Purgo Biologics, Challans, France) mixed with I-PRF (Figure 6C). Moreover, the bone defect required horizontal augmentation; therefore, additional material was placed on the buccal side of the defect and covered with collagen membrane Osseoguard Flex (Zimmer Biomet Dental, Biomet 3i, Palm Beach Gardens, FL, USA) fixed with osseofixation plates and closed with 5-0 nylon sutures (Figure 6D–F).

**Figure 7:**
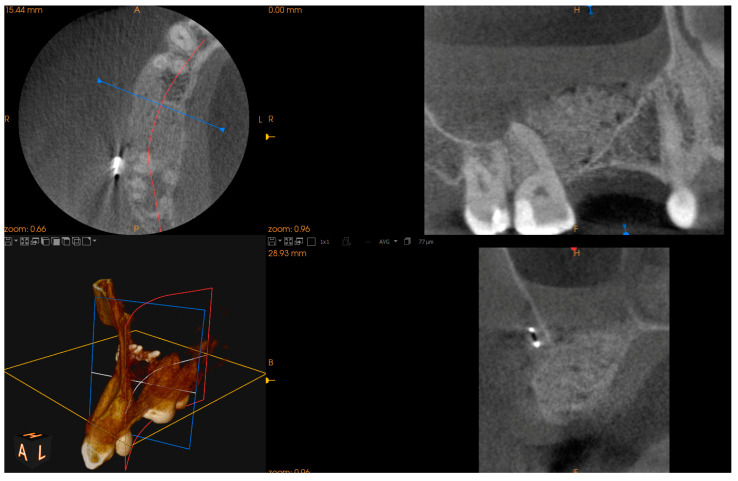
Screenshot from post-op CBCT scans with visible xenograft and a flat level of liquid in the maxillary sinus lumen due to present perforation.

Post-operation CBCT scan shows properly compacted material within the sinus and on the side of the alveolar ridge (Figure 7). No material migration into the lumen of the sinus was observed.

After the procedure, the patient was prescribed the following:

- Amoxicillin with clavulanic acid 875 + 125 mg every 8 h for 9 days;
- Azithromycin 500 mg every 24 h for 6 days, first-day dose is 1 g (double);
- Probiotic every 8 h;
- Ibuprofen 400 mg every 6 h, in case of pain;
- Nimesulide 100 mg every 12 h, in case of pain;
* Xylometazoline in spray 0.05% (0.5 mg/mL), administered during inhale, aiming straight up the nose, 3 doses per side every 8 h;
* Mometasone in spray 0.05% (50 µg/dose); twice a day, although about 10 min after the xylometazoline.

* recommended as a precautionary measure in case of BAV = Bad Anatomical Variant, which means narrow (lesser than 5 mm diameter) ostium of the maxillary sinus.

The patient was provided the following postoperative instructions:

- It is absolutely forbidden to do the following:
  ○ Blow one’s nose;
  ○ Use cold packs (due to additional lateral augmentation);
  ○ Strain the cheek to look over (as it may damage the sutures);
  ○ Check by touching/pressing with the finger;
- It is strongly advised not to sneeze, especially by grasping the nose. It is allowed to sneeze with the mouth wide open;
- If needed, rinse very gently, without excessive pressure. It is allowed to rinse by slowly turning one’s head side to side and letting the water/mouthwash flow by itself;
- If a patient uses any denture, it is forbidden to wear it for at least 4 months post-op;
- It is disadvised to use a sauna or swimming pool for 2 months;
- Any dynamic/cardio sports are also disadvised. Yoga or stretching is allowed;
- The diet should be diverse and soft, although not necessarily blended;
- It is recommended to sleep on the opposite side (from the operated one) with the head placed higher than the rest of the body, i.e., on 2–3 pillows. Any blood stains on the sheets are considered a good sign, as the sinus ostium is unobstructed;
- First check-up in the clinic 48–72 h after the procedure.

After 6 months, the alveolar ridge height was suitable for implant placement. No postoperative complications during the 8-year post-op period were reported.

**Figure 8:**
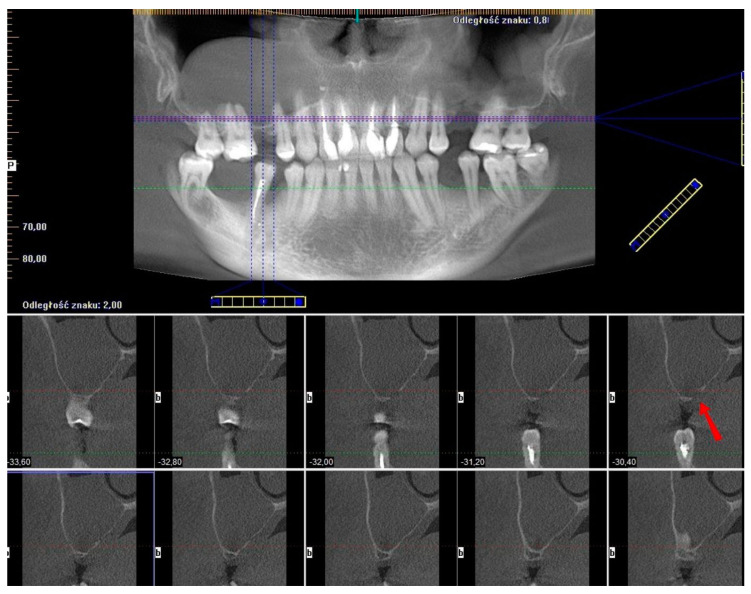
Croco Eye Technique—final version. Screenshot of the pre-op CBCT scans with a visible radiopaque lesion in the right maxillary sinus, insufficient alveolar ridge height, and noncontinuous bone of the sinus floor due to past oroantral communication (OAC) (red arrow).

The process of removing the cyst’s lining poses a threat to the excessive widening of the Scheiderian membrane’s perforation. Generally, this type of complication requires enlarging the osteotomy window in order to avoid further perforation widening during membrane elevation. However, in certain cases, i.e., when teeth roots restrict the maximum size of the osteotomy window, shredding the Schneiderian membrane and progressing perforation is highly undesirable. Such difficulties may be restrained with a slight modification of the primary version of the CET, as presented in the second case, where only tooth 16 was missing (Figure 8).

### 2.2. Case 2—Final Version

Local anesthesia and premedication were identical to those in the first case.

**Figure 9:**
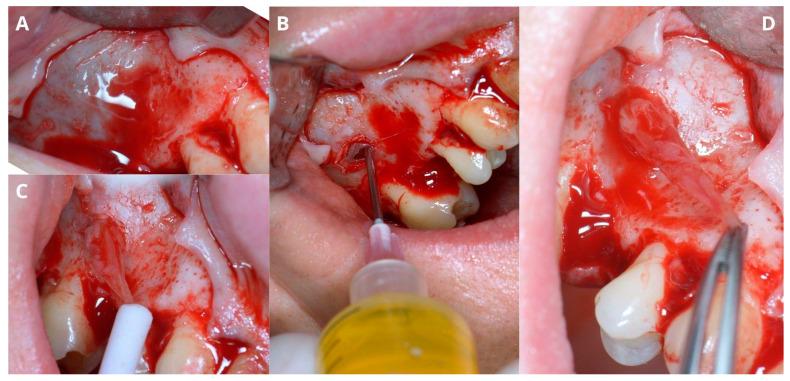
Preparing for the first osteotomy. Elevation of a full-thickness flap (**A**). Creating a smaller window and aspirating the retention cyst’s yellow content (**B**). Removal of the cyst’s lining, firstly with narrow suction (**C**), then gently with tweezers (**D**). The perforation will not enlarge more than the diameter of osteotomy, as the periosteum is still attached to the bone, therefore remains safe.

**Figure 10:**
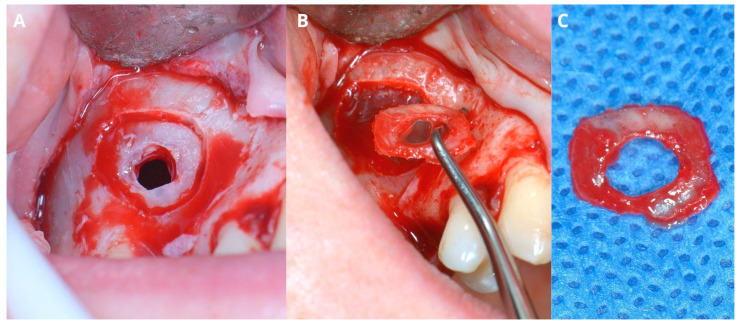
Creating the second, larger window around the perforation (**A**). Detachment of the bony ring from the Schneiderian membrane (**B**) and the ring itself (**C**).

After elevating the mucoperiosteal flap (Figure 9A), a minor, about 3–5 mm wide osteotomy window is opened simply to allow biopsy (Figure 9B) and remove the cyst’s lining (Figure 9C,D). The perforation will not exceed the size of the minor window as the Schneiderian membrane remains safely attached to the bone. Afterward, a second, major osteotomy window is made around the minor one (Figure 10A). The existing perforation should be located in the center of the major window. Then, the bone ring is gently elevated and detached from the Schneiderian membrane (Figure 10B,C). Elevation should start from the center of the ring, not from its margin, since if the new perforation appears on the sidelines, it will be almost impossible to manage properly. From this point, the whole procedure is similar to the one described above: elevating the Schneiderian membrane, managing its perforation, and completing the augmentation.

**Figure 11:**
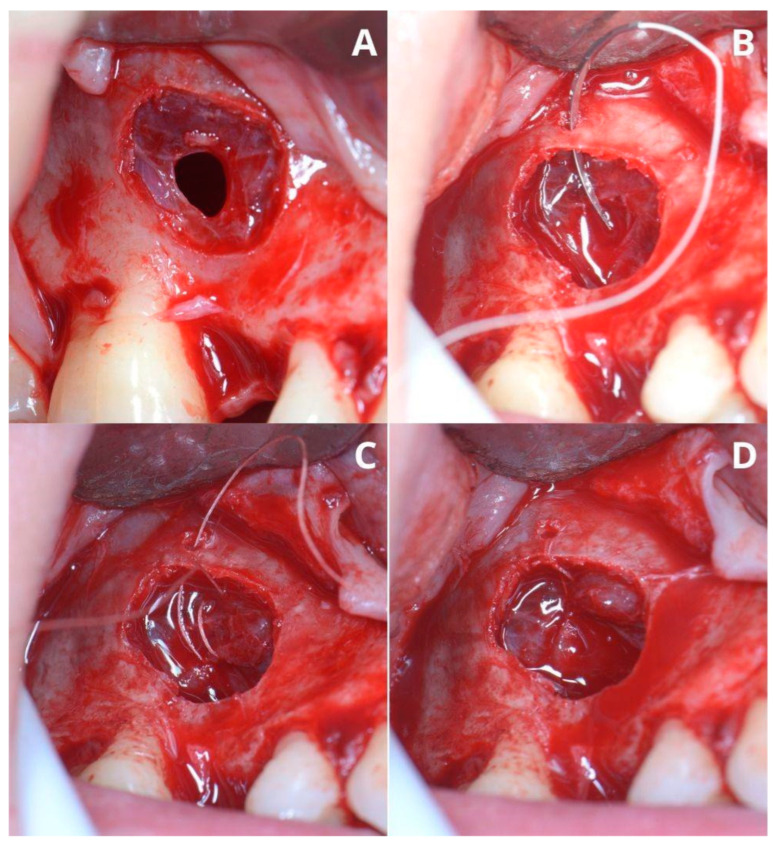
Managing the Schneiderian membrane perforation. The perforation is located in the center of the bigger window (**A**). The needle of the resorbable PGA (polyglycolic acid) 5-0 suture gets through a tiny hole (which was previously drilled) in the upper edge of the window (**B**). A double loop closes the perforation (**C**), and the knot is made around the tiny bone hole (**D**).

**Figure 12:**
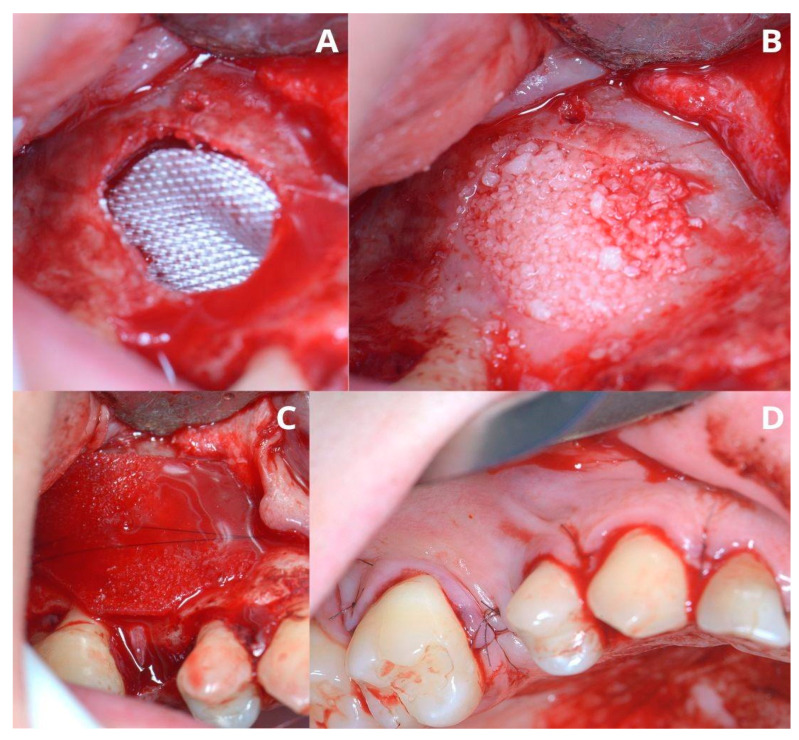
Completing the sinus lift. Oxycellulose was put directly under the Schneiderian membrane (**A**), then xenografted with I-PRF (**B**) and closed with collagen membrane (**C**). The horizontal suture (5-0 nylon) is meant to hold the collagen membrane in place and prevent the xenograft from migrating buccally to the soft tissues. The wound was closed with 5-0 nylon sutures (**D**).

**Figure 13:**
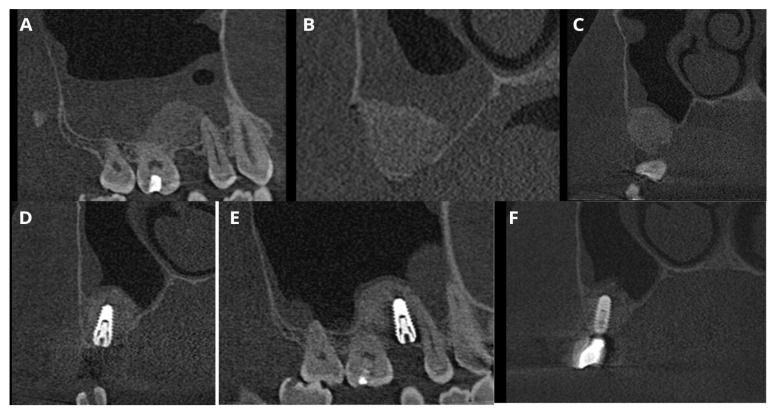
Screenshots of the post-op CBCT scans. Immediately after Croco Eye (**A**,**B**), 6 months post-op: before (**C**) and after implant placement (**D**,**E**). The latest follow-up was 5 years 2 months post-op with a loaded implant (**F**). Throughout the follow-up period, no recurrence of the cyst was detected.

In this case, after removing the osseous ring, the Schneiderian membrane perforation was closed with resorbable polyglycolic acid 5-0 suture (Figure 11) and BloodSTOP™ iX (LifeScience Plus, Mountain View, CA, USA) (Figure 12A) before placing the xenograft with I-PRF (Figure 12B). Nevertheless, the authors find suturing Schneiderian perforation difficult and threatening to advance the perforation dimensions; hence, it is inadvisable for inexperienced surgeons. Xenograft was covered with a collagen membrane Osseoguard Flex (Zimmer Biomet Dental, Biomet 3i, Palm Beach Gardens, FL, USA) and closed with 5-0 nylon sutures (Figure 12C,D). Figure 13 presents post-op CBCT screenshots and radiological follow-up.

In this case, identical instructions and medications were provided. During the 5-year post-op period, nopostoperative complications were reported. All patients provided verbal and written consent for the treatment procedures.

### 2.3. Case 3—Final Version Upgraded with a Surgical Guide for the Window Osteotomy

**Figure 14:**
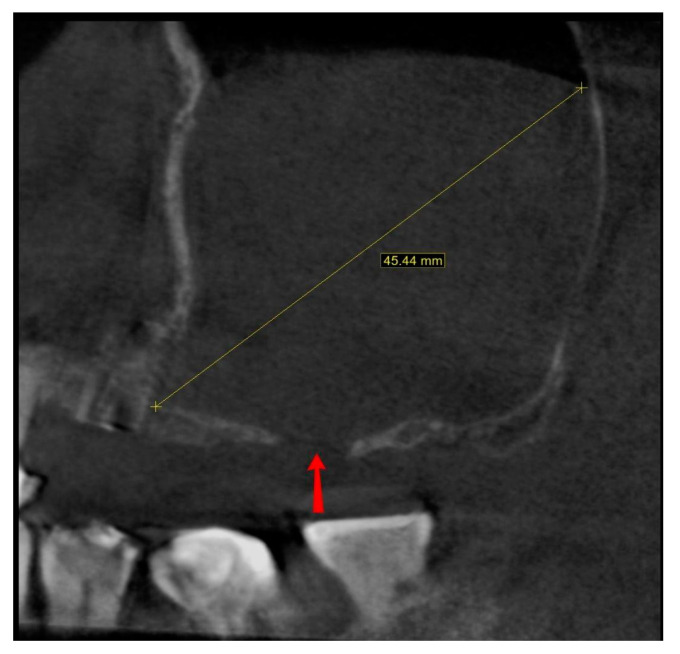
Screenshot from the pre-op CBCT scan showing maximal retention cyst’s diameter (45.44 mm; yellow line). Also, the past oroantral communication window is visible (red arrow).

**Figure 15:**
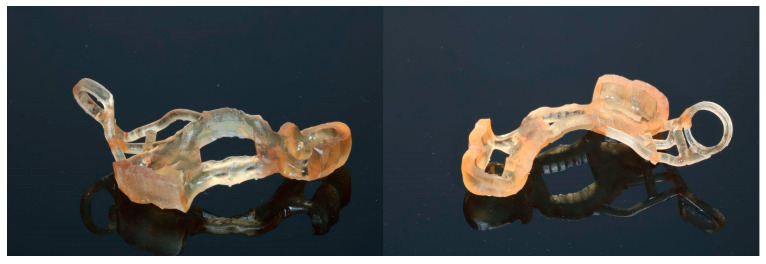
A 3D-printed surgical guide was created to simplify the osteotomy and to determine an optimal location for placing a xenograft during sinus lift.

**Figure 16:**
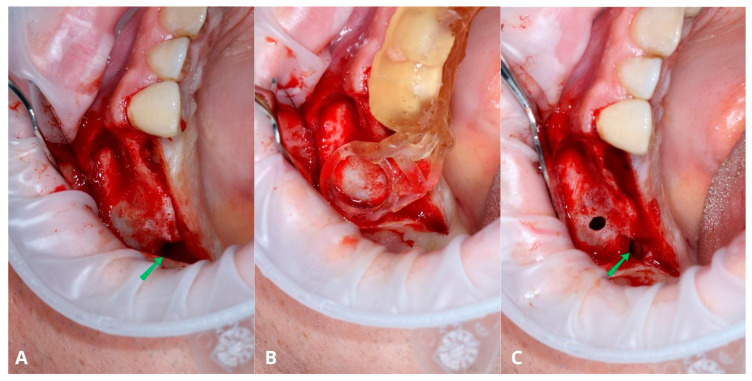
Full-thickness flap elevation with an oroantral communication (OAC) (green arrow) visible from the very beginning of the surgery (**A**). The rounded part of the surgical guide indicates the most optimal location of the osteotomy from the augmentation point of view (**B**). Smaller osteotomy after removal of the cyst’s lining (**C**). The OAC is also visible here (green arrow).

The last presented case (by A.N.) is the variation of the final version of the CET. It shows effectiveness even against a large (45.44 mm maximum diameter) retention cyst (Figure 14). Moreover, the sinus lift part was guided by a surgical guide (Figure 15), which indicated the ideal position of the window. The decision to conduct the surgery with a guide was dictated by the potential risk of the nonoptimal location of the osteotomy due to a lack of teeth or other reference points in that area. Lastly, this case presented another difficulty in the form of past oroantral communication (OAC) (Figure 16), which, from the very beginning, sentenced the surgeon to deal with another perforation of the Schneiderian membrane.

**Figure 17:**
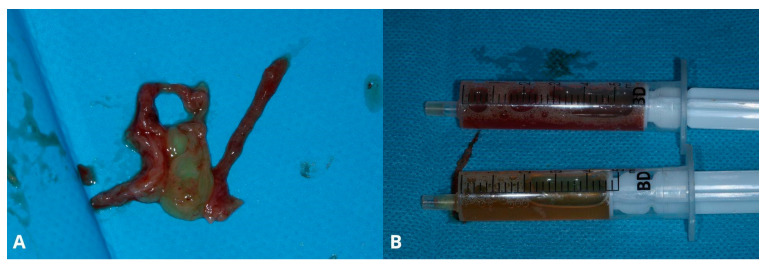
The extracted cyst’s lining (**A**) and yellowish content were drawn with syringes (**B**).

**Figure 18:**
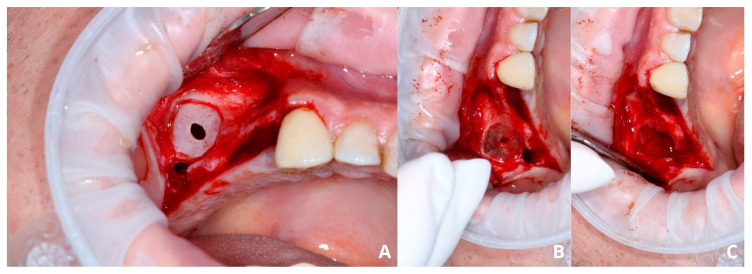
The second osteotomy was created around the first one (**A**). Situation after the removal of the bony lid (**B**) and after the elevation of the Schneiderian membrane with visible resorbable suture (**C**).

**Figure 19:**
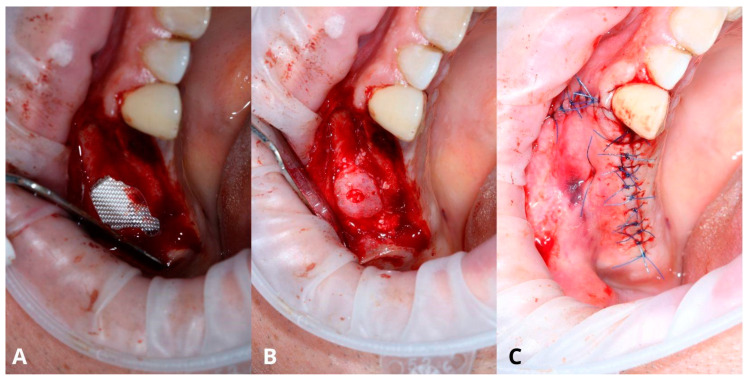
Oxycellulose was placed just under the Schneiderian membrane (**A**) and then xenografted with I-PRF, collagen, and A-PRF membranes. The bony lid was put back in place (**B**) and later stabilized with a mattress suture. Final closure with 5-0 nylon sutures (**C**).

The main flow of the surgery remained the same as in the final version of the Croco Eye Technique: creating a smaller window, aspirating a cyst’s content, removing its lining, and completing the sinus lift (Figure 16, Figure 17, Figure 18 and Figure 19). There were two perforations (one after the cyst’s removal and the second after OAC), and the decision was made to manage them by suturing with a 7-0 resorbable PGA suture.

**Figure 20:**
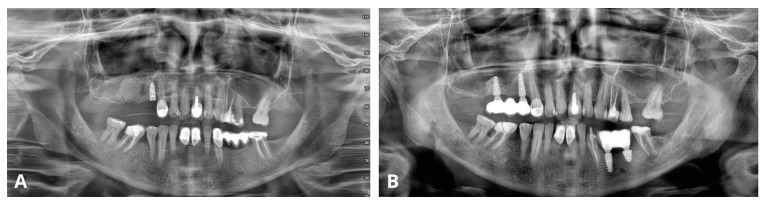
Comparison of orthopantomogram (OPG) immediately after the procedure (**A**) and 4 years after delivering the final reconstruction on implants (**B**).

The last figure (Figure 20) compares OPG radiographs after the procedure and 4 years after the patient received the bridge on implants, showing no recurrence of the cyst and stable implants with prosthetic reconstruction.

### 2.4. Clinical Data

These three patients set proper examples of the Croco Eye Technique and provided detailed photographic documentation even in difficult cases. The outcomes of this technique may be divided into primary and secondary. The primary intraoperative outcome was a Schneiderian membrane’s perforation, which sometimes may progress uncontrollably. The primary postoperative outcome was the possibility of placing implants in the augmentation area due to the successful elimination of the retention cyst and performance of the sinus lift. The secondary outcomes were definitive elimination of the retention cyst and a successful reconstruction of implants.

The potential cyst’s recurrence was established via CBCT analysis: the presence of any radiological image typical for retention cysts was qualified as the original one’s recurrence, even though it could be a new cyst that grew in the same region. The success of implant treatment was confirmed by the lack of any signs of inflammation or periimplantitis in the area of implants (both radiologically and in the clinical examination) and by the lack of discomfort reported by the patient.

This study is an important contribution to the sinus lift topic; all the more, there is a lack of similar long-term follow-up cases described [2]. Throughout the years, this technique has been successfully implemented on multiple patients. Cases conducted by three researchers (R.J., A.N., and K.O.) are summarized in Table 1.

## 3. Results

This study summarizes 33 cases of CET, 9 of the primary version (27.27%) and 24 of the final version (72.73%). There were 18 males (54.55%) and 15 females (45.45%), and the average age of a patient was 49.33 years (minimum 29, maximum 79 years).

The average height of a retention cyst was 24.05 mm, with a minimum value of 12 mm and a maximum of 40.2 mm. In 8 cases (24.24%), some sort of anatomical difficulties were reported, among which the most common was past oroantral communication (4 cases). Uncontrolled perforation happened six times (five times in the primary version and one time in the final version). The prevalence of this complication is 55.56% in the primary version and 4.17% in the final version.

The perforation was sutured with resorbable sutures eight times (24.24%), although it was not always connected with uncontrolled shredding of the Schneiderian membrane, and sometimes it was added just as an extra precaution.

Intraoperative hemorrhage was another reported complication, which occurred in two cases (6.06%). One of them might have been connected to an artery in the lateral wall of the sinus.

The average alveolar ridge height was 1.86 mm, with a minimal height of 0.7 mm and a maximum height of 3.9 mm. In three cases (9.09%), implants were placed immediately during the procedure.

In all 33 cases (100%), a chosen bone graft was xenogenic, and in 26 cases (78.79%), some sort of blood centrifuged derivative was added to the material (PRP, I-PRF, or L-PRF). This took place in 5 cases (55.56%) of the primary version and 21 cases (87.5%) of the final version.

In one case (3.03%), the post-op CBCT revealed graft dislocation to the sinus lumen. As only part of the material migrated, the following control after 6 months showed sufficient bone volume for placing implants, even though it was not an optimal and desired amount. The graft’s migration was probably connected to the uncontrolled shredding of the Schneiderian membrane and lack of suturing of the perforation. This case was also a primary version of the CET.

One patient disappeared after the procedure and did not show up for follow-up and continuation of treatment; therefore, they were excluded from the “follow-up part” of the statistics.

In one case (3.13%), a cyst’s recurrence was reported 6 months after the procedure. This can be explained by the fact that the lining could not be extracted during the procedure and remained in the sinus. Such a situation happened twice (6.06%), although in the second case, the recurrence was not observed. Moreover, in these two cases, the histopathological examination could not be conducted due to a lack of appropriate material, and the retention cysts were diagnosed based on the yellowish color of the aspirated content and radiological image.

Of 28 patients (87.5% of the initial group) that continued with implantation and their prosthetic reconstruction, in all 28 of them, the treatment was successful (100% implant survival). This verdict was based on the right radiographic image on CBCT, the lack of any negative symptoms reported from the patients and inflammation signs, and general satisfaction with treatment. One patient desired to change the prosthetic reconstruction to a new one with a lighter color, although it was purely a matter of aesthetics, and here, it is not classified as a lack of success. Four patients (12.5%) are still undergoing the treatment process (marked with a “-” in Table 1).

The follow-up period was, on average, 48.625 months (slightly above 4 years), with a maximum of 110 months (9 years and 2 months) and a minimum of 6 months. The follow-up period was counted from the point of CET surgery until the last CBCT. The average follow-up for the primary version was 81 months, and for the final version, 35.96 months.

For some patients, this value could have been higher, as their last visit to the clinic was a few years ago.

## 4. Discussion

### 4.1. Differentiation

At the beginning, a mucous retention cyst and a mucocele (of the maxillary sinus) must be distinguished, as they are commonly mistaken in the literature. Mucous retention cysts develop under the mucosa layer of the Schneiderian membrane, which explains why their lining is so thin [2,3]. Mucocele’s cystic wall is thicker [8] and exhibits osteolytic properties. It occupies the whole sinus space and closes its ostium, while mucous retention cysts are non-expanding and delicate, usually located on the floor of the sinus [8,9]. A pseudocyst is similar to a retention cyst radiographic image and thus can be easily mistaken, although histopathological examination proves it does not have a lining [2].

As retention cysts are fairly common lesions of maxillary sinuses with a prevalence between 3.5 and 10.1% [10,11] up to 28.6% [1,12], it should be deliberated whether or not they must be removed or whether they increase any risk of complications for the sinus lift procedure.

A study by Kim et al. [13] shows that the presence of retention cysts did not have a negative impact on the survival rate of sinus lift or implants.

As described by Testori et al. [7], the sole presence of a retention cyst is not a contradiction for sinus elevation, although they could become problematic. When elevated to the level of the ostium, they may close the ostium [7,14] (due to post-intervention swelling and continuous growth of a cyst [15]) and block the sinus drainage, which most likely will result in postoperative sinusitis and failure of the augmentation procedure [7]. Moreover, elevating the Schneiderian membrane with full cysts may result in its piercing and contamination of the augmentation material [16].

### 4.2. Surgical Tactics and Timing

Testori et al. offer two different treatment options [7]. The first is an endoscopic approach before the sinus lift, which is usually performed by an otolaryngologist (ENT specialist). The second is the removal or marsupialization of the retention cyst by aspirating the cyst’s contents during sinus lift. The authors of that study followed 15 patients who underwent treatment by intraoperative content aspiration. In 12 of them (80%), cysts disappeared, while in the remaining 3 (20%), CBCT scans showed the presence of reduced cysts; therefore, the sole aspiration of contents cannot be considered a curative treatment [16]. Eliminating the possibility of recurrence of the residual cyst may be achieved by removing its lining [9].

Simultaneous sinus lift with cyst removal not only reduces the number of surgical procedures and overall treatment period but also enables its histopathological examination.

A recent study by Lee et al. [15] addresses different sinus lesions (pseudocyst and two sizes of retention cyst) and how to perform sinus lift and/or implant placement in these conditions. In the case of a pseudocyst, spontaneous drainage was conducted, although no sample was taken for histopathological examination; therefore, a diagnosis of pseudocyst was presumed solely by a radiographic image. As for mucous retention cysts, a smaller cyst (<20 mm) was just aspirated, and no sample was retrieved for histopathological examination, so the diagnosis was based only on the radiological image and yellowish color of the aspirated fluid. In the case of a bigger retention cyst (>20 mm), the patient earlier underwent a Caldwell–Luc operation, and afterward, during the second surgery, when the cyst content was aspirated, the lining was examined (retention cyst was confirmed), and the implant was placed, though without bone substitute placement. The authors of that study suggest different strategies for different sizes of lesions; however, the CET addresses all the above-mentioned situations in a comprehensive manner by delivering a universal technique suitable even for difficult cases of bigger cysts. Moreover, it enables safe retrieval of the cyst’s lining and subsequent histopathological examination to confirm the diagnosis and preclude recurrence.

Also, a study by Choi et al. [17] describes different approaches for different sizes of retention cysts. Nevertheless, the CET is universal for all sizes of mucous retention cysts, which is much easier for the clinician to remember and implement.

In a study by Han et al. [18], a retention cyst was aspirated, and implants were immediately placed without bone augmentation. This approach may be promising if the residual alveolar ridge height is sufficient to obtain primary stability of the implants (in the above-mentioned study, bone height varied between 3.5 and 7.5 mm). In cases of lesser bone height, when sinus lift with a lateral window approach is required, the CET seems to be a safe and more predictable solution.

A study by Berberi et al. [4] describes retention cyst removal with immediate sinus lift and implant placement. They present a similar approach as described in this article, although the aspiration (and subsequently perforation) was located near the edge of the bony window, therefore posing a risk of a progressive tearing of the Schneiderian membrane during its elevation. The final version of CET addresses this issue by designing two osteotomies.

In a study by Lin et al. [19], although it describes removing the pseudocyst, not the retention cyst, the authors suggest waiting 3 months before sinus lift and describe their method as time-shortening, in comparison to Caldwell–Luc operation and standard 6 to 12 months of healing of the respiratory ciliated epithelium. Our study also shows that the CET enables shortening of the overall treatment period, and instead of waiting even 18 months (12 after standard Caldwell–Luc and 6 after sinus lift), implants can be placed immediately with the cyst’s excision.

Another very recent study by Testori et al. [14] also approaches this topic and highlights that there is no standard treatment protocol, and clinical decisions vary from ignoring the retention cyst (or pseudocyst) to conducting a Caldwell–Luc operation or endoscopy and then waiting 6 months for the epithelium to properly heal. That study describes a method of aspirating the content of the retention cyst without removing its lining and, therefore, without making a perforation in the Schneiderian membrane (if delicately conducted). This solution demonstrates both advantages and disadvantages and thus needs to be properly analyzed. Retrieving the cyst’s lining enables its histopathological examination to properly confirm the diagnosis and lowers the risk of the cyst’s recurrence. On the other hand, removing the lining means creating the Schneiderian membrane perforation. The authors agree that perforations happen even in simple, classical sinus lift procedures, and either way, the clinician needs to be able to manage the perforation. If properly fixed, it has no negative impact on the final outcome [7]. The final version of the CET addresses this issue by providing a way to minimize the risk of its uncontrolled widening. In the end, removal of the lining has a beneficial effect of lowering the chance of cyst recurrence and is believed to have more positive aspects than just aspiration of the cyst’s content.

At last, a similar study and surgical approach was described by Chiapasco and Palombo in 2015 [20]. They also present the removal of a cyst (pseudocyst, in their case) through a smaller window and continuing sinus lift through a bigger window, although it is shown that the smaller window is above the bigger one, not within. Their case series contains 12 patients with a mean follow-up of 50 months, which we consider sufficient time for presenting long-term results. The presented technique enables the avoidance of Schneiderian membrane perforation, which is a solid advantage and may be more suitable for less-experienced surgeons. This technique is definitely worth considering.

### 4.3. Schneiderian Membrane’s Perforation

As a result of the cyst’s excision, the clinician has to deal with a Schneiderian membrane perforation. When the periosteum underneath the cyst has lost the mucosa of the Schneiderian membrane, it becomes weaker; therefore, even the small perforation may progress easily [16].

In the primary version of CET, the very act of extracting the lining may lead to tearing and uncontrolled widening of the perforation, even up to 15 mm [21], which could be particularly unfavorable, especially in cases where the surgeon has limited options of managing this type of complication, i.e., due to anatomical features.

In the data presented above, in more than half (55.56%) of primary version cases, the perforation shredded uncontrollably. The final version of CET assumes extracting the cyst’s lining through the narrow window, which enables the operator to keep the Schneiderian membrane’s perforation safely limited to the window’s diameter, which led to reducing the uncontrolled perforation to 4.17%. Afterward, a second, wider window is cut to a size suitable for comfortable sinus floor elevation and proper placement of the material.

Repairing a perforation during a sinus lift procedure depends on its size and location [7]. In general, minor perforations can be left for self-repair by clot formation or foldover. Larger perforations need repairing after elevating the Schneiderian membrane but before placing the augmentation material. Perforations larger than 5 mm may be repaired by a resorbable membrane that retains its shape. Soft and shapeless membranes (when wet) would not play their role and are not recommended [7]. Another way to repair a perforation is suturing loose, torn Schneiderian membrane with resorbable sutures to the small holes drilled in the lateral wall of the maxillary sinus, as shown in the second case (Figure 11). Furthermore, compressed PRF clots—rich in platelets, leucocytes, cytokines, and growth factors—can be pieced together, as they show resilience and adequate adhesiveness to repair large perforations [7,21,22]. In our sample, 78.79% of cases were conducted with PRP/I-PRF/L-PRF to improve healing and help restrain the granules of the graft from migrating.

The authors suggest combining these methods by dipping thick shape-retaining membranes in I-PRF to benefit from both solutions. Suturing was also implemented, although it requires advanced surgical skills and, in inexperienced hands, may result in greater, torn perforation. Among our patients, suturing with resorbable sutures was implemented in 8 cases, which was 24.24% of all cases. Also, for better adhesion, BloodSTOP™ iX (LifeScience Plus, Mountain View, CA, USA) was used. Ineffective perforation repair leads to material infection and its displacement, which affects the whole treatment outcome [21].

The implant success rate throughout the group of patients who attended follow-up appointments was 100%, although this group was limited to only 28; therefore, in a bigger sample, this rating might become lower. The authors believe that the number of analyzed cases is the main limitation of this study.

This type of surgical intervention needs to be thoroughly analyzed, and a CBCT requires assessing the level of difficulty of a given case [23] in comparison to clinicians’ skills and experience so that the procedure will be conducted as smoothly and safely for the patient as possible.

Eventually, a two-stage surgery can be conducted, with separate lesion excision and, after a few months, when the decreased swelling of the sinus membrane is confirmed by CBCT scans, delayed sinus lift [16]. This postponed approach combined with endoscopic excision techniques is strongly advised if the “dome-shaped” lesion is not, in fact, a retention cyst but a soft tissue mass or tumor. In such cases, fine-needle aspiration cytology (FNAC) can be conducted via the CET approach. Moreover, when using the presented technique, a second attempt at an open sinus lift will not cause great problems due to the minor size of the opened window.

## 5. Conclusions

The Croco Eye Technique is best suited for a mucous retention cyst excision in patients who need an open sinus lift procedure before implant placement. Implementation of this technique results in Scheiderian membrane perforation, although it enables immediate implantation. Sole aspiration of the cyst’s contents may result in its recurrence and cannot be considered a curative treatment; hence, simultaneous removal of the cyst’s lining is advised, as the recurrence rate was only 3.13%. The problem of uncontrolled Schneiderian membrane perforation may be unraveled by creating two windows: smaller for cyst excision and larger for sinus lift procedure. Schneiderian membrane perforation may be repaired by using shape-retaining collagen membranes combined with I-PRF and resorbable sutures. If conducted properly, it has no negative impact on the survival rate of implants, which reached 100%. The Croco Eye Technique is not suitable for removing solid tumors, although it enables access for fine-needle aspiration cytology (FNAC) if needed.

## Figures and Tables

**Table 1:** Clinical data of 33 cases of the Croco Eye Technique. For better clarity, the cases of the primary version are highlighted in light orange, whereas that of the final version are highlighted in light green. The symbol “-” means that a patient is still undergoing the treatment process.

No.	The Primary or Final Version of CET	Sex	Age	Maximum Height of Residual Cyst (in mm)	Other Anatomical Difficulties (i.e., Septa, Lateral Wall Artery in the Window Area, Oroantral Communication (OAC))	Uncontrolled Schneiderian Membrane’s Perforation?	Other Clinical Information	Crestal Bone Height (in mm)	Immediate Implantation?	Type of Graft (xeno/allo +/− PRF)	Graft Dislocation to the Sinus Lumen (On Post-op CBCT Scans)?	The Success of the Sinus Lift (After 6 Months)?	Cyst’s Recurrence (After 6 Months)?	Successful Implants with Restoration (CBCT 12 Months after Implantation)?	Follow-Up (In Months; from the CET until the Last Check-Up with CBCT)
1	primary	F	55	26.5	no	no		1.48	no	xeno (porcine) + collagen membrane + I-PRF	no	yes	no	yes	98
2	primary	M	48	19.4	no	no		3.6	yes	xeno (porcine) + collagen membrane + I-PRF	no	yes	no	yes	84
3	final	M	43	27.0	palatal wall window after OAC	no (+ resorbable 5-0 suturing)		1.2	no	xeno (porcine) + collagen membrane + I-PRF	no	yes	no	yes	62
4	final	F	42	13.7	no	no		1.35	no	xeno (porcine) + collagen membrane + I-PRF	no	yes	no	yes	15
5	final	M	37	34.4	no	no	cyst’s lining could not be extracted	2.2	no	xeno (porcine) + collagen membrane + I-PRF	no	yes	no	-	9
6	primary	M	42	18.0	crestal ridge after OAC	yes (+ resorbable 4-0 suturing)	pinning the collagen membrane to the crestal bone 5× Ti pins	0.8	no	xeno (porcine) + collagen membrane + PRP	no	yes	no	yes	73
7	primary	M	37	34.3	no	yes (+ resorbable 6-0 suturing)	fixing bone “lid” back with an osseofixation plate	1.74	no	xeno + collagen membrane	no	yes	no	yes	49
8	final	M	54	40.2	crestal ridge after OAC	no (+ resorbable 7-0 suturing)	max. cyst diameter 45.44 mm; fixing bone “lid” with mattress sutures	1.19	no	sticky bone xeno (porcine) + L-PRF	no	yes	no	yes	47
9	final	M	30	12.0	no	no (+ resorbable 6-0 suturing)	collagen membrane pinned 5× Ti pins	1.5	no	xeno + collagen membrane	no	yes	no	yes	13
10	primary	F	54	30.4	no	yes (+ resorbable 6-0 suturing)		3.9	no	xeno + collagen membrane	no	yes	no	yes	52
11	final	M	45	22.1	palatally septa	yes (+ resorbable 6-0 suturing)	collagen membrane pinned 5× Ti pins	0.9	no	xeno (porcine) + collagen membrane	no	yes	no	-	6
12	final	M	44	19.8	no	no		2.4	no	xeno (porcine) + collagen membrane	no	yes	no	yes	28
13	final	F	35	24.7	no	no		0.7	no	xeno (porcine) + collagen membrane + I-PRF	no	yes	no	yes	37
14	primary	F	61	14.9	no	yes		1.57	no	xeno (porcine) + collagen membrane	no	yes	no	yes	78
15	final	M	29	23.2	no	no		1.6	no	xeno (porcine) + collagen membrane + I-PRF	no	yes	no	yes	64
16	final	F	43	16.2	no	no		2.9	no	sticky bone xeno (porcine) + collagen membrane + L-PRF	no	yes	no	yes	52
17	final	F	50	16.8	mesio-lateral septa	no		3.45	yes	sticky bone xeno (porcine) + collagen membrane + L-PRF	no	yes	no	yes	22
18	final	F	49	32.8	no	no		1.84	no	xeno (porcine) + collagen membrane + I-PRF	no	yes	no	-	10
19	final	M	68	25.5	no	no		3.9	yes	xeno (porcine) + collagen membrane + I-PRF	no	yes	no	yes	19
20	final	M	54	14.1	lateral wall artery in the window area	no	intraoperative hemorrhage	1.13	no	xeno (porcine) + collagen membrane + I-PRF	no	yes	no	yes	44
21	final	F	37	18.0	no	no		1.04	no	xeno (porcine) + collagen membrane + I-PRF	no	yes	no	yes	46
22	primary	M	71	29.4	mesio-lateral septa	yes		1.58	no	xeno (porcine) + collagen membrane	yes (partial)	yes (lesser volume)	no	yes	82
23	final	F	66	18.6	no	no		2.15	no	xeno (porcine) + collagen membrane + I-PRF	no	yes	no	yes	51
24	final	M	48	36.2	no	no	cyst’s lining could not be extracted	1.83	no	xeno (porcine) + collagen membrane + I-PRF	no	yes	yes	yes	70
25	final	M	55	31.5	no	no		1.4	no	xeno (porcine) + collagen membrane + I-PRF	no	no data	no data	no data	no data
26	final	F	54	21.3	no	no	intraoperative hemorrhage	1.67	no	xeno (porcine) + collagen membrane + I-PRF	no	yes	no	yes	24
27	final	M	39	24.6	no	no		2.8	no	sticky bone xeno (porcine) + L-PRF	no	yes	no	-	14
28	final	M	32	35.4	crestal ridge after OAC	no (+ resorbable 5-0 suturing)		1.59	no	xeno (porcine) + collagen membrane + I-PRF	no	yes	no	yes	47
29	final	F	64	17.3	no	no		1.17	no	xeno (porcine) + collagen membrane + I-PRF	no	yes	no	yes	46
30	final	M	58	30.7	no	no		2.45	no	xeno (porcine) + collagen membrane + I-PRF	no	yes	no	yes	69
31	final	F	60	25.2	no	no		2.02	no	sticky bone xeno (porcine) + L-PRF	no	yes	no	yes	32
32	primary	F	79	16.6	no	no		1.76	no	xeno (porcine) + collagen membrane + PRP	no	yes	no	yes	110
33	primary	F	45	22.9	no	no		1.48	no	xeno (porcine) + collagen membrane + PRP	no	yes	no	yes	103

## Data Availability

The data presented in this study are available on request from the corresponding author. The data are not publicly available due to privacy issues.

## Abbreviations

CBCT	cone beam computed tomography
CET	Croco Eye Technique
FNAC	fine-needle aspiration cytology
OAC	oroantral communication
OPG	orthopantomogram
PGA	polyglycolic acid
PRF	platelet-rich fibrin
